# Analytical Model for the Channel Maximum Temperature in Ga_2_O_3_ MOSFETs

**DOI:** 10.1186/s11671-021-03490-6

**Published:** 2021-02-10

**Authors:** Xiaole Jia, Haodong Hu, Genquan Han, Yan Liu, Yue Hao

**Affiliations:** State Key Discipline Laboratory of Wide Band Gap Semiconductor Technology, School of Microelectronics, Xidian University, Xi’an, 710071 China

**Keywords:** Analytical model, Maximum temperature, Ga_2_O_3_, Thermal conductivity

## Abstract

In this work, we proposed an accurate analytical model for the estimation of the channel maximum temperature of Ga_2_O_3_ MOSFETs with native or high-thermal-conductivity substrates. The thermal conductivity of Ga_2_O_3_ is anisotropic and decreases significantly with increasing temperature, which both are important for the thermal behavior of Ga_2_O_3_ MOSFETs and thus considered in the model. Numerical simulations are performed via COMSOL Multiphysics to investigate the dependence of channel maximum temperature on power density by varying device geometric parameters and ambient temperature, which shows good agreements with analytical model, providing the validity of this model. The new model is instructive in effective thermal management of Ga_2_O_3_ MOSFETs.

## Background

Gallium oxide (Ga_2_O_3_)-based metal–oxide–semiconductor field-effect transistors (MOSFETs) are excellent candidates for next generation power electronics, which are benefited from two major advantages of Ga_2_O_3_: the significantly high bandgap (~ 4.8 eV) and high-quality bulk crystals produced at low cost [1]. Tremendous efforts have been devoted to improving its electrical properties in all aspects like current density [2], breakdown voltage [3], and power figure-of-merit [4]. With the experimental confirmation of its unprecedented potential for power electronic devices [5–9], it is now of paramount importance to explore the performance and reliability of Ga_2_O_3_ MOSFETs, such as the issue of self-heating effects and hence the channel maximum temperature (*T*_max_), due to its relatively low thermal conductivity (*κ*, 0.11–0.27 Wcm^−1^ K^−1^ at room temperature) [1].

In recent years, various methods for estimating the *T*_max_ of Ga_2_O_3_ MOSFETs have been proposed theoretically and experimentally [10–13]. In general, numerical simulations can quantitatively estimate *T*_max_ of a certain device. However, this is time consuming [14]. On the other hand, the extraction of *T*_max_ through experiments is always underestimated [15]. Therefore, an analytical model has to be made in order to adequately model the *T*_max_ in Ga_2_O_3_ MOSFETs, which can provide sufficient accuracy with time-efficiency and qualitative assessments [14].

In this paper, we propose an analytical model of *T*_max_ for Ga_2_O_3_ MOSFETs by employing Kirchhoff’s transformation, considering the dependence of *κ* on temperature and crystallographic directions for Ga_2_O_3_. The proposed model can be applied for Ga_2_O_3_ MOSFETs with native or high-thermal-conductivity substrates. The validity and the accuracy of the analytical model are methodically verified by comparison with the numerical simulations via COMSOL Multiphysics.

## Methods and Model Development

**Fig. 1:**
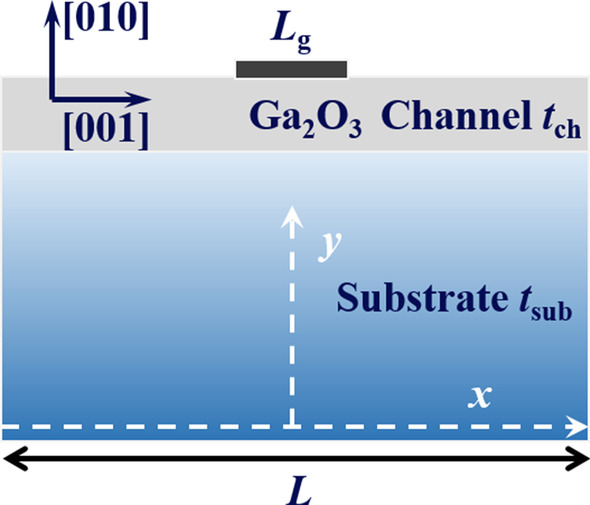
The schematic diagram of Ga_2_O_3_ MOSFET

The analytical model for *T*_max_ in Ga_2_O_3_ MOSFETs is proposed based on the structure shown in Fig. 1. Key parameters of structure are listed in Table 1. In fact, it has been demonstrated that Joule heating is concentrated at the drain edge of the gate in Ga_2_O_3_ MOSFETs [13]. In order to simply the model, it is assumed that the heating effect from the gate is uniform [12] and can completely penetrate through the gate oxide due to its negligible thickness. Different substrate materials underneath Ga_2_O_3_ channel are considered in this model, such as bulk Ga_2_O_3_ and high *κ* materials, aiming at the board feasibility and compatibility. Thus, the device is viewed as a two-layer problem. The substrate contacts with an ideal heat sink so that the bottom surface is isothermal, and its temperature equals to that of ambient temperature (*T*_amb_, 300 K by default). Adiabatic boundary conditions were imposed on other surface of the structure. These boundary conditions can be summarized as [14, 16]

**Table 1:** Key parameters of structure

Symbol	Quantity	Default value
*L*_g_	Gate length	2 μm
*L*	Device length	150 μm
*t*_ch_	Channel thickness	300 nm
*t*_sub_	Substrate thickness	500 μm

1 $${\kappa }_{y}{\left.\frac{\partial T}{\partial y}\right|}_{y={t}_{ch}+{t}_{sub}}=\left\{\begin{array}{c}\frac{P}{{L}_{g}} \left|x\right|\le \frac{{L}_{g}}{2}\\ 0 \left|x\right|>\frac{{L}_{g}}{2}\end{array}\right.,$$

2 $${\left.T\right|}_{y=0}={T}_{amb},$$

3 $${\left.\frac{\partial T}{\partial x}\right|}_{x=-\frac{L}{2}}={\left.\frac{\partial T}{\partial x}\right|}_{x=\frac{L}{2}}=0,$$

where *P*, *T* and *κ*_y_ denote the power dissipation density, temperature and thermal conductivity of [010] direction for Ga_2_O_3_, respectively. It should be emphasized that the unit of *P* is W/mm in this paper.

The *κ* value of Ga_2_O_3_, one of the key parameters for the thermal characteristic of material, plays an important role in the diffusion of heating effect as well as the accuracy of model. That is to say, a carefully description of *κ* value is required, due to its serious anisotropy and temperature-dependence [17]. In general, the dependence of *κ* of Ga_2_O_3_ on temperature (*T*) along two different crystal orientations ([001] and [010]) is given by

4 $${\kappa }_{\left[001\right]}\left(T\right)=0.137\times {\left(\frac{T}{300}\right)}^{-1.12},$$

5 $${\kappa }_{\left[010\right]}\left(T\right)=0.234\times {\left(\frac{T}{300}\right)}^{-1.27}.$$

The comparison study of *T*_max_ at different *P* was carried out by COMSOL Multiphysics, considering constant and realistic *κ*, respectively. We found that at a *P* of 1 W/mm, *T*_max_ values of 533 K and 622 K are obtained, respectively (not shown). Therefore, it is quite necessary to take into account the impacts of *T* and crystallographic direction on the *κ* of Ga_2_O_3_ in the model.

**Fig. 2:**
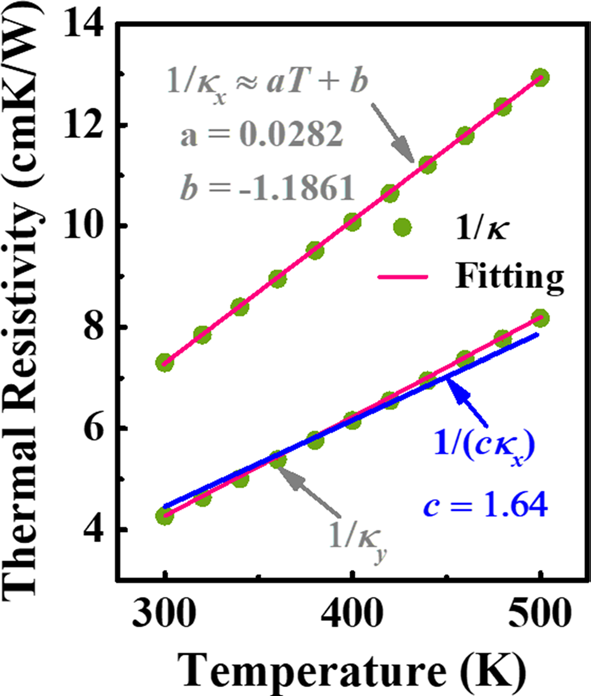
The relationship between the thermal resistivity and *T* for directions of [001] and [010]. Green symbols and red lines denote actual and fitted values, respectively. Blue line represents the hypothesis of 1/*κ*_y_ ≈ 1/(*cκ*_x_), where *c* = 1.64

The temperature behavior is governed by the heat conduction equation. The heat conduction equation at steady-state in Ga_2_O_3_ domain is

6 $$\frac{\partial }{\partial x}\left({\kappa }_{x}\left(T\right)\frac{\partial T}{\partial x}\right)+\frac{\partial }{\partial y}\left({\kappa }_{y}\left(T\right)\frac{\partial T}{\partial y}\right)=0,$$

where *κ*_*x*_ denotes the thermal conductivity of [001] direction for Ga_2_O_3_. The nonlinear heat conduction equation can be solved by employing Kirchhoff’s transformation. However, the application of Kirchhoff’s transformation may be restricted due to the highly anisotropic *κ* in Ga_2_O_3_, which is valid, strictly speaking, only for materials with isotropic *κ* [14]. Given the above limitation, one should not consider *κ*_*x*_ and *κ*_*y*_ to be two independent variables. Figure 2 shows the relationship between the thermal resistivity, i.e., 1/*κ*, and *T* for directions of [001] and [010] over a large *T* range, respectively. It can be seen that 1/*κ*_*y*_ can be substituted with 1/(*cκ*_*x*_) and *c* is chosen to be equal to 1.64. Consequently, Eq. (6) can be transformed to the following equation:

7 $$\frac{\partial }{\partial \mathrm{x}}\left({\kappa }_{x}\left(T\right)\frac{\partial T}{\partial x}\right)+\frac{\partial }{\partial \mathrm{y}}\left({c\kappa }_{x}\left(T\right)\frac{\partial T}{\partial y}\right)=0.$$

Based on the preceding approximations of *κ*_*x*_ and *κ*_*y*_, the Kirchhoff’s transformation can be employed without any restrictions. Besides, it also can be seen that the reciprocal of *κ* is expected to be proportional to *T.* Thus, in order to reduce the computational complexity, the expression of 1/*κ*_*x*_ can be simplified as 1/*κ*_*x*_ = *aT* + *b*, as shown in Fig. 2. The reason for the use of *a*, *b* and *c* is just convenience in writing the equations that follow.

By the application of Kirchhoff’s transformation and the method of separation of variables, the expression of *T*_max_ can be derived as

8 $$\begin{aligned} T_{{max}} = & \\ & \,\left( {T_{{amb}} + \frac{b}{a}} \right)exp\left( {\frac{{aP\left( {t_{{ch}} + t_{{sub}} } \right)}}{{cL}} + \frac{{aPSL}}{{\sqrt c \pi ^{2} L_{g} }}} \right) - \frac{b}{a}, \\ \end{aligned}$$

where

9 $$S=\sum_{n=1}^{\infty }\frac{\mathrm{sin}n\pi \frac{{L}_{g}}{L}}{{n}^{2}}\frac{\mathrm{sinh}2n\pi \frac{{t}_{ch}+{t}_{sub}}{\sqrt{c}L}}{\mathrm{cosh}2n\pi \frac{{t}_{ch}+{t}_{sub}}{\sqrt{c}L}}.$$

It should be pointed out that *S* is a convergent infinite series and its approximate value which can be obtained easily is used in calculation instead of its actual value.

In the case of Ga_2_O_3_ MOSFETs with high *κ* substrates, Kirchhoff’s transformation cannot be directly applied theoretically. In fact, for the transformation to be valid, the boundary conditions should be either isothermal (constant *T* surface), or have a fixed heat flux density. However, due to the different *κ* of Ga_2_O_3_ and substrate material, both of these boundary conditions are not completely met at the Ga_2_O_3_/substrate interface. Considering that the *κ* of Ga_2_O_3_ is much lower than high *κ* substrate, a hypothesis, the isothermal interface between the Ga_2_O_3_ and the substrate, is introduced. This hypothesis is instrumental in deriving the expression *T*_max_ and its validity will be verified later. In this case, the thermal resistance (*R*_TH_) of high *κ* substrate, a ratio of the difference between the *T*_int_ and *T*_amb_ and the *PW*, i.e., *R*_TH_ = (*T*_int_—*T*_amb_) / (*PW*), can be calculated as *R*_TH_ = *LW*/(*κt*_sub_), where *W* is the width of substrate [19]. Thus, the expression of the temperature of Ga_2_O_3_/substrate interface (*T*_int_) is

10 $${T}_{int}=\frac{P{t}_{sub}}{{\kappa }_{sub}L}+{T}_{amb},$$

where *κ*_sub_ is the thermal conductivity of heterogeneous substrate, which is assumed to be constant. In addition, it should be pointed out that the thermal boundary resistance between Ga_2_O_3_ and heterogeneous substrates is not included in the model. Therefore, with the help of Eq. (8), the expression of *T*_max_ for Ga_2_O_3_ MOSFETs with heterogeneous substrate can be derived as

11 $$\begin{aligned} T_{{max}} = & \\ & \;\left( {T_{{int}} + \frac{b}{a}} \right)exp\left( {\frac{{aPt_{{ch}} }}{{cL}} + \frac{{aPSL}}{{\sqrt c \pi ^{2} L_{g} }}} \right) - \frac{b}{a}, \\ \end{aligned}$$

where

12 $$S=\sum_{n=1}^{\infty }\frac{\mathrm{sin}n\pi \frac{{L}_{g}}{L}}{{n}^{2}}\frac{\mathrm{sinh}2n\pi \frac{{t}_{ch}}{\sqrt{c}L}}{\mathrm{cosh}2n\pi \frac{{t}_{ch}}{\sqrt{c}L}}.$$

## Results and Discussion

**Fig. 3:**
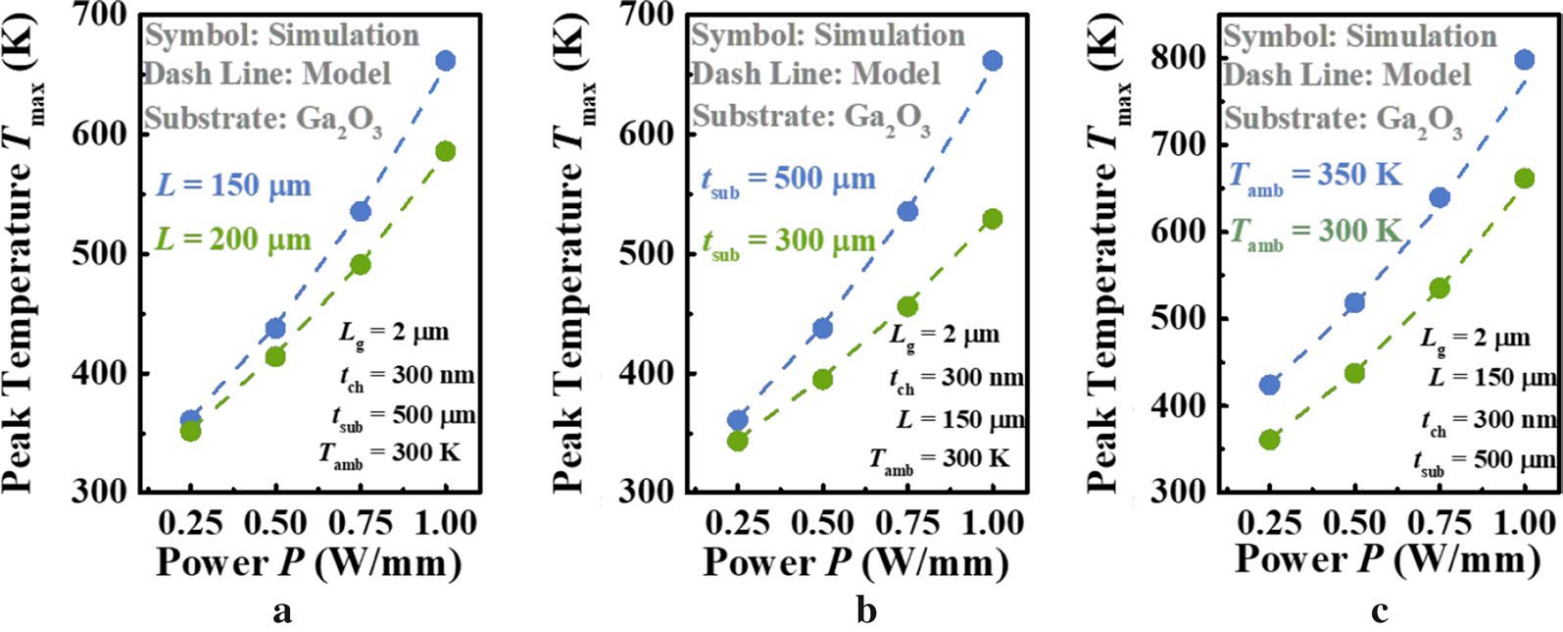
Dependence of *T*_max_ on **a** the length of device *L*, **b** the thickness of substrate layer *t*_sub_, and **c** ambient temperature *T*_amb_ at different power *P*. Symbols and lines denote the results of proposed model and simulation, respectively

The validity of the analytical model for the *T*_max_ in Ga_2_O_3_ MOSFETs was systematically verified in this section, considering both native substrate and the counterpart with higher thermal conductivity. The best way to test the validity of a model is against experimental data. However, some key geometric parameters could not be found in experimental literatures, such as *t*_sub_ and *L* in Ref. [12]. Therefore, finite-element simulation, one of the most accurate means, is used to verify our model. Figure 3 shows dependence of *T*_max_ on power density *P* obtained from both COMSOL Multiphysics and analytical model, for Ga_2_O_3_ MOSFET with native substrate. Varied key parameters are considered, including device length *L*, substrate thickness *t*_sub_, and ambient temperature *T*_amb_. As shown in Fig. 3a, the *T*_max_ is naturally increased with the raised power density and the increase rate is boosted with the smaller *L*. This is attributed to that the device with larger *L* allows heat dissipation from the active region and hence its overall temperature is lower than that with smaller *L* at same *P* [11]. That is to say, its *R*_TH_, the slope of curves, is smaller than that of latter*.* Furthermore, since the *κ* of Ga_2_O_3_ will decrease with the increase in overall temperature, its *R*_TH_ will also increase slower than that with smaller *L* consequently, which is obvious in Fig. 3a [19]. Similarly, the investigation of dependence of *T*_max_ on *t*_sub_ was performed, as illustrated in Fig. 3b. It is observed that the trend of *T*_max_ with respect to *P* is same as that in Fig. 3a. The thinner substrate always produces the alleviated rise in *T*_max_ over the enlarged power density, which is understandable that the thinner substrate, the lower overall temperature, the smaller *R*_TH_ and its increase rate, just like the analysis in Fig. 3a. Figure 3c compares the influence of *T*_amb_ on *T*_max_ as *P* increases. It is evident that the difference between two curves increases slowly, which is different from those in Fig. 3a, b. Ordinarily, *R*_TH_ is dominated by the geometric parameters of device and the *κ* value of material. However, considering that the structure is fixed in this case, the increase in *R*_TH_ is only induced by the decrease in *κ* of Ga_2_O_3_. On the other hand, a high level of agreement is observed for the proposed model, which considers the *T*- and direction-dependent relationship for the *κ* of Ga_2_O_3_, confirming the scalable nature of the model. On average, the difference of proposed model and simulation is < 1 K. The overall excellent agreement observed suggests that the proposed model is highly effective and accurate.

**Fig. 4:**
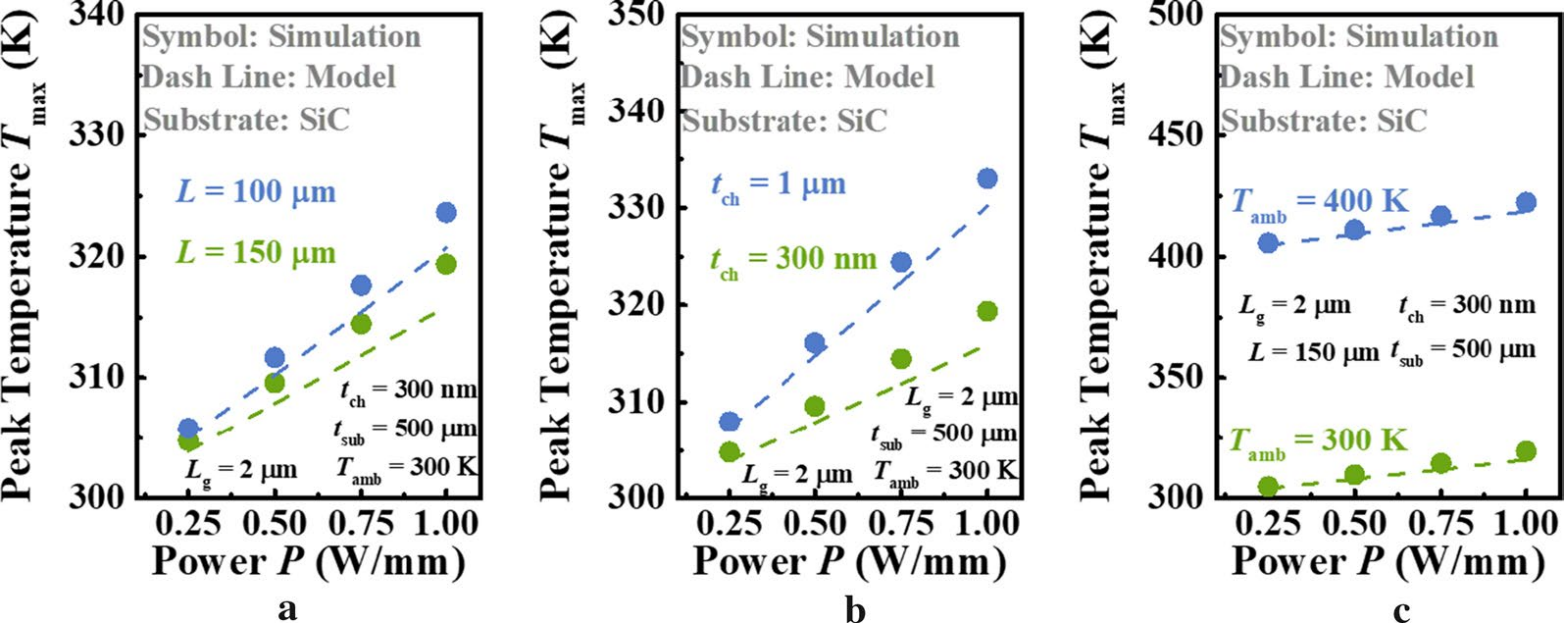
Dependence of *T*_max_ of Ga_2_O_3_ MOSFETs with SiC substrate on **a** the length of device *L*, **b** the thickness of Ga_2_O_3_ layer *t*_ch_, and **c** ambient temperature *T*_amb_ at different power *P*. Symbols and lines denote the results of proposed model and simulation, respectively

**Fig. 5:**
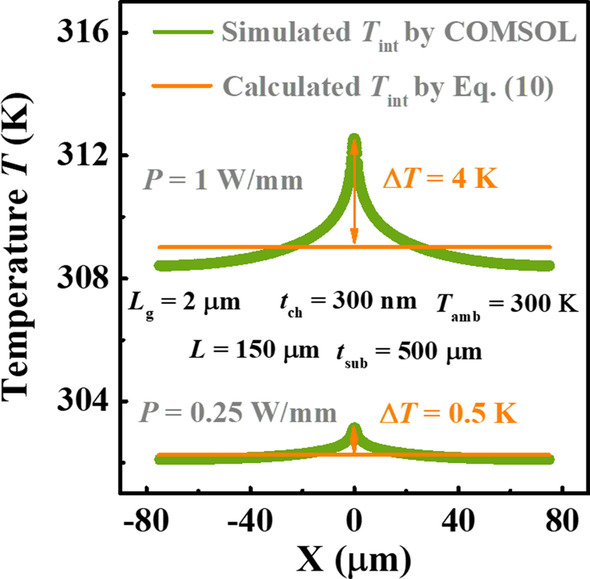
Comparison of *T*_int_ between simulated and calculated by Eq. (10) at different *P*

Likewise, as shown in Fig. 4, the similar comparisons are repeated for Ga_2_O_3_ MOSFETs on high *κ* substrate, SiC. Here, the steps for *L* and *T*_amb_ that we choose are larger than those in Fig. 3, and the varied channel thickness *t*_ch_ is considered instead of *t*_sub_ in this case. Otherwise, the difference between two curves of *T*_max_ with respect to *P* in each figure will be undistinguishable, owing to the efficient heat dissipation capacity of SiC substrate. The *κ* of SiC (3.7 Wcm^−1^ K^−1^) applied is a default parameter in COMSOL Multiphysics software. Thanks to high *κ* of SiC, it can be seen clearly from all figures that the increase in *T*_max_ is approximately linear as *P* increases, which means that the influence of temperature on the *R*_TH_ of device is negligible. It should be pointed out that our model can describe this linear relationship successfully. However, it is obvious that the *T*_max_ calculated by current model is lower than that predicted by simulation, and this difference is more evident with the increase in power consumption. To show this mechanism, simulated *T*_int_ are extracted with the power increasing and compared with calculated *T*_int_ by Eq. (10) as plotted in Fig. 5. It is found that the Joule heating becomes more concentrated in the middle of device as *P* increases. There are 0.5 K and 4 K Δ*T* between the model and simulation at this location when *P* = 0.25 and 1 W/mm, respectively. This is the reason that our model fails to accurately predict *T*_max_. Therefore, a more reasonable hypothesis of *T*_int_ is needed to obtain higher accuracy in future. Nevertheless, the *T*_max_ is predicted by model to be only < 4 K lower than that by simulation even under 1 W/mm power dissipation density. That is to say, although the hypothesis of uniform *T*_int_ is inconsistent with fact, our model can provide an estimation of *T*_max_ with enough accuracy.

## Conclusions

An accurate analytical model to estimate the *T*_max_ of Ga_2_O_3_ MOSFETs involving the temperature- and direction-dependent of thermal conductivity is presented. A simple expression based on device geometry and material parameters has been derived. An excellent agreement has been obtained between the model and COMSOL Multiphysics numerical simulations by varying different power consumption. The proposed model for the *T*_max_ is of great importance for effective thermal management power devices especially Ga_2_O_3_ MOSFETs.

## Data Availability

The datasets supporting the conclusions of this article are included within the article.

## Abbreviations

Ga_2_O_3_: Gallium Oxide
MOSFETs: Metal–oxide–semiconductor field-effect transistors
AlGaN: Aluminum Gallium Nitride
GaN: Gallium Nitride
SiC: Silicon Carbide
